# Sorbent track: Quantitative monitoring of adsorbed VOCs under in-situ plasma exposure

**DOI:** 10.1038/srep31888

**Published:** 2016-08-24

**Authors:** Zixian Jia, Antoine Rousseau

**Affiliations:** 1 LPP, Ecole Polytechnique, UPMC, CNRS, Université Paris-Sud 11, 91128 Palaiseau Cedex, France

## Abstract

Sorbent-TRACK is a new device developed to monitor adsorption and surface oxidation of pollutants under direct plasma exposure. It is based on direct transmitted Fourier Transformed Infrared (FTIR) spectroscopy. A pyrex reactor under controlled gas pressure and composition is inserted on the infrared beam of a commercially available Nicolet 5700 FTIR spectrometer. A substrate holder is located on the optical path of the infrared beam. A thin pellet of a dedicated catalyst (CeO_2_ in the present work) is inserted in a substrate holder and can be exposed to direct plasma treatment using a Dielectric Barrier Discharge. The time resolution of Sorbent-TRACK is limited by the time resolution of the Nicolet 5700 FTIR spectrometer and close to 30 s. The dynamic of the adsorption and plasma oxidation of acetone and isopropanol on CeO_2_ are studied and intermediates are monitored. Performances and sensitivity of Sorbent-TRACK are reported Adsorption and oxidation of acetone leads to production of adsorbed isobutene and acetic acid, where oxidation of isopropanol gives mainly to adsorbed acetone, mesityl oxide and acetate. An increase of the plasma power leads to an increase of the isopropanol and acetone oxidation rate and a related increase of the production of adsorbed intermediates.

The combination of a Non-Thermal Plasma with a catalyst or sorbent has been studied for many years for various applications such as exhaust gas purification (soot oxidation and NO_x_ reduction)^1^, Volatile Organic Compounds (VOC) removal for indoor air purification^2^ and more recently for CO_2_ valorization^3^,^4^.

Indoor air purification is a major health issue as well as a rising market. In 1983, World Health Organization has defined Sick building syndrome (SBS), usually reported by occupants in certain buildings or specific rooms^5^,^6^. Sources located inside the building, such as adhesives, carpeting, wood products and cleaning products may emit VOCs^7^. Techniques for VOCs control in exhaust air streams include such as adsorption^8^. thermal and catalytic oxidation^9^, and photocatalysis^10^. Such methods may be cost-inefficient and difficult to operate when low concentrations of VOCs need to be treated in indoor air, typically lower than 1 ppm^8^.

Non-thermal plasma (NTP) is an effective way to produce oxidative species in air, at ambient temperature and at a low energy cost^11^,^12^,^13^. Energetic electrons generated in non-thermal plasmas can collide with carrier gases, forming highly reactive species such as free radicals and excited atoms, molecules and ions. When combined with a catalytic sorbent, a NTP triggers some surface oxidative reaction of adsorbed VOCs. In some cases, synergetic effects are observed in the sense that, for example, the removal rates obtained with a plasma-catalytic setup are higher than those predicted by simply adding the effects of plasma and catalyst^2^,^14^. The combination of NTP with heterogeneous catalysts can be divided into two categories depending on the location of the catalyst: *in-situ* plasma catalysis (IPC) and post-situ plasma catalysis (PPC). The latter is a two-stage process where the catalyst is located downstream of the plasma reactor, while the former is a single-stage process with the catalyst being exposed to the active plasma.

Numerous studies have been dedicated to studying the performances of Plasma-Catalyst coupling. However, to the day, most studies have focused on the gas phase analysis; the destruction of an injected VOC is reported and often a carbon balance is calculated. Only few articles have reported a dynamic monitoring of species adsorbed by infrared on the catalytic surface under plasma exposure (IPC^15^,^16^ or PPC^17^,^18^).

Infrared spectroscopy (IR) is for that purpose very appropriate since it is fast and sensitive. In a recent paper Barakat *et al*.^18^ investigated the evolution of adsorbed phase species by post-situ plasma regeneration (PPC) using Diffuse Reflectance Infrared Fourier Transform Spectroscopy (DRIFTS). It has been proven that the complete oxidation of isopropanol and acetone is mainly limited by the acetone oxidation rate. DRIFT spectroscopy was coupled to conventional gas phase analysis using FTIR. A reaction scheme was proposed for explaining the formation of those adsorbed intermediates and gaseous products. Similarly, Sauce *et al*.^17^ studied acetaldehyde catalytic ozonation in the post-situ plasma-catalysis process^19^ in which, an acetaldehyde saturated Ag/TiO_2_/SiO_2_ surface^20^ was brought into contact with a NTP. These studies show that combining monitoring of the gas phase and of the surface greatly improves the understanding of surface oxidation mechanisms.

Two recent papers have monitored adsorbed species in in-plasma configuration PPC: Rivallan *et al*.^16^ process by operando FTIR spectroscopy when the catalyst is in direct contact with a Dielectric Barrier Discharge (DBD-IR reactor). Measurements were combined with a DBD on an Al_2_O_3_ surface to enable *in situ* and time resolved monitoring of the oxidation of pre-adsorbed IPA. Although no quantitative concentration could be given, very interesting oxidative mechanisms could be deduced. Stere *et al*.^15^ reported a newly developed DRIFT-MS system for the investigation of non-thermal plasma (NTP) assisted heterogeneously catalyzed reactions, when a plasma jet is used in contact with a Ag/Al_2_O_3_ catalytic surface. The results provided further evidence of the role of NTP in promoting the performance of the Ag catalyst at low reaction temperature. These works emphasize that *in situ* monitoring developments are possible and necessary.

However, so far, quantitative monitoring of the surface has not yet been published in the case of the In-plasma configuration, when the plasma is in direct contact with the sorbent/catalytic surface.

A newly developed system Sorbent-TRACK dedicated to quantitative analysis of adsorbed species on various sorbents under direct plasma exposure (IPC). In the present paper, test pollutants are isopropanol (IPA) and acetone. The catalyst is CeO_2_ which is considered as an effective promoter in thermal catalytic reactions due to its high oxygen storage capacities and redox properties between Ce^4+^ and Ce^3+^. CeO_2_ can also act as a local source/sink of oxygen species for due to its high bulk oxygen mobility and oxygen vacancies^21^.

In the present work, a two-step protocol is followed to investigate surface reactions: i) First, the adsorption of the test pollutants on CeO_2_ is monitored without plasma; reactive adsorption products may also be identified. ii) then, the gas phase inlet of the test pollutants is turned off and the plasma is turned on; NTP oxidation of the test pollutants adsorbed on ceria is monitored as well as adsorbed oxidation intermediates.

## Experimental Set Up

### Sorbent-TRACK cell

**Figure 1:**
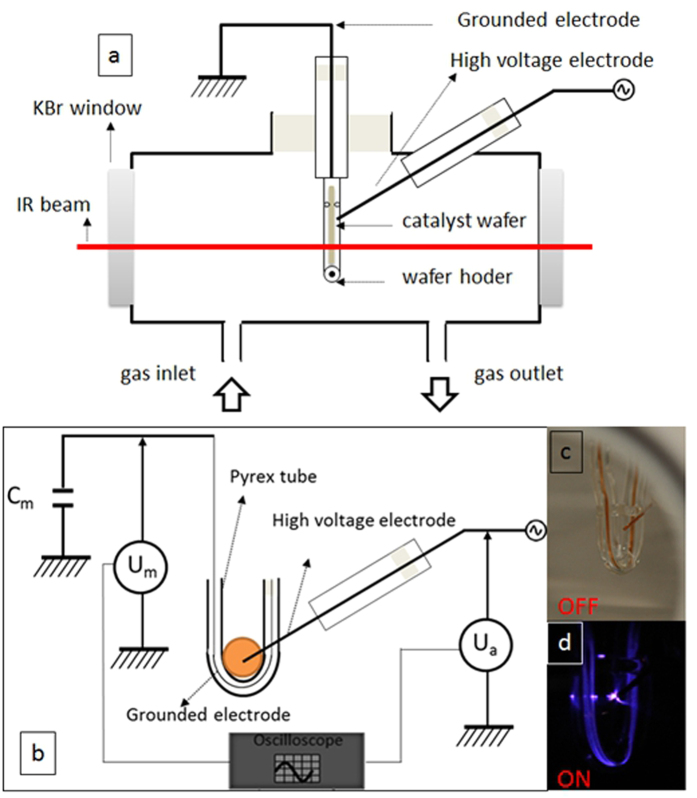
(**a**) Longitudinal view of sorbent track cell (Grounded electrode and high voltage electrode is copper wire; Catalyst wafer is placed in the IR pathway fixed by wafer holder of Pyrex tube.); (**b**) Electrical circuit of the *in-situ* DBD reactor (Pyrex tube is the insulating dielectric barrier); Photo of catalyst wafer holder of Plasma off (**c**) and on (**d**).

The Sorbent-TRACK cell as shown in Fig. 1a consists of a cylinder of glass carrying a toroidal sample holder in its center, where the catalyst is placed in the form of a self-supported wafer. The heating system guarantees a maximum temperature of 573 K on the sample. The tightness can be obtained by using Kalrez O-rings between the terminal KBr windows and the extremities of the cell. The plasma reactor consists of a Pyrex glass tube of 5.8 mm inner diameter, 8 mm outside diameter. The inner electrode (H.V.) consists of a copper wire of 30 μm and the outer electrode is a copper wire of 30 μm placed in the center of sample holder. The electrical parameters (U_a_, U_m_) were measured via two high voltage probe (LeCroy, PPE20KV-CC) connected to a digital oscilloscope (LeCroy WaveSurfer 64Xs-A, 600 MHz). A measurement capacitance (Cm) of 680 pF is placed in series with the DBD reactors. The equivalent electrical scheme is represented in Fig. 1b.

### Catalytic materials and gas flow set-up

Catalytic CeO_2_ powder with a specific surface of 84.41 ± 5.32 m^2^/g was pressed into self-supported wafers (Ø = 7 mm, m ∼60 mg cm^−2^. thickness of 0.3 mm); experiments were carried after activation of the pellet at 473 K for 2 h and then cooling down to room temperature.

The VOCs in this study are 2-Propanol (34959 – CHROMASOLV^®^, for HPLC, absolute, 99.9%) and acetone (270725 – CHROMASOLV^®^, for HPLC, ≥99.9%) both prepared by Sigma–Aldrich. Certified gas cylinders are supplied by Air Liquide. The regulation of the gas flow was insured using Brooks mass flow controllers. Synthetic air was used to prepare the carrier gas flow.

**Figure 2:**
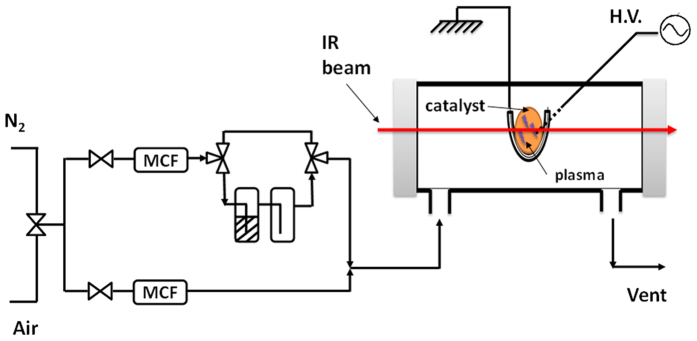
Schematic representation of experimental setups of the gas injection and the Sorbent-TRACK.

The Sorbent-TRACK cell is connected to a flow set-up, as shown in Fig. 2. Gases are introduced into the lines by mass flow controllers. A two way valve allows the selection of either air or N_2_ as the main carrier gas. The flow is then divided, into two gas lines, one of which is the main flow and the other through the cryostat containing the liquid VOC. The flowmeter attributed to the main gas flow can go up to 2000 mL/min (±1%, i.e. is less accurate below an imposed flow of 20 mL/min) while the one used for VOC dilution has a maximum of 10 mL/min and can be used accurately up to 0.1 mL/min. The temperature of cryostat and the flow through the cryostat were respectively 0 °C and 5 ml/min for IPA and −0 °C and 3.9 ml/min for acetone. The main flow was adjusted to have a total flow of 500 ml/min.

### Spectra acquisition using FTIR spectroscopy

IR spectra were collected with a Nicolet 5700 FTIR spectrometer equipped with a MCT detector. The spectra have been treated by the Nicolet OMNIC software. Two spectra per minute are collected with Omnic software with 16 scans per spectrum and a spectral resolution of 0.5 cm^−1^. For calibration curves and quantification, TQ Analyst 8 from thermo scientific was used.

Spectra of species adsorbed on the catalytic samples are acquired as follow: the infrared beam emitted by the FTIR source is collimated through the entrance window of Sorbent-TRACK, propagates through the catalytic sample, and exits though the exit window to be detected on the MCT detector.

Alternatively, Sorbent-TRACK may be replaced by a 10 m optical-path White cell to calibrate the concentration of VOCs in the gas phase. In these conditions (no catalytic samples), the detection limits of this analytical tool have been determined as two times the signal/noise ratio in the region of interest and are: 80 ppb for acetone, 90 ppb for IPA, 20 ppb for CO_2_, 10 ppb for CO and 15 ppb for O_3_.

### Plasma generation

**Figure 3:**
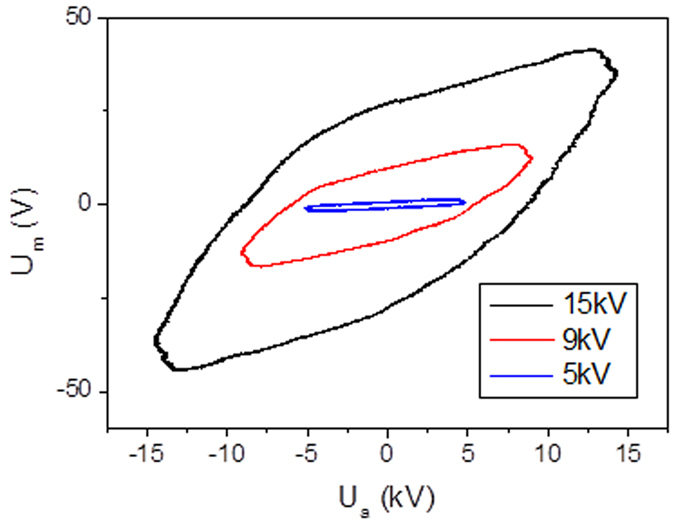
Example of a Lissajous figure used to calculate the injected power. It is obtained by applying a voltage of 14 kV, 9 kV and 5 kV respectively and a frequency of 50 Hz in a typical DBD reactor with a Cm of 680 pF. under air flow by means of a sinusoidal power supply.

The injected power is obtained by the Lissajous figures^22^, corresponding to the plotting of the transported electric charge through the discharge as a function of the applied periodical voltage. Experimentally, the charge is delivered from the voltage drop across the reactor and the average electric energy dissipated in a discharge cycle is the area of the characteristic Lissajous figure. Figure 3 shows such Lissajous figures obtained by applying a voltage of 15 kV, 9 kV and 5 kV respectively and a frequency of 50 Hz in a typical DBD reactor (Fig. 1c,d, respectively off and on) under air flow by means of a sinusoidal power supply.

**Figure 4:**
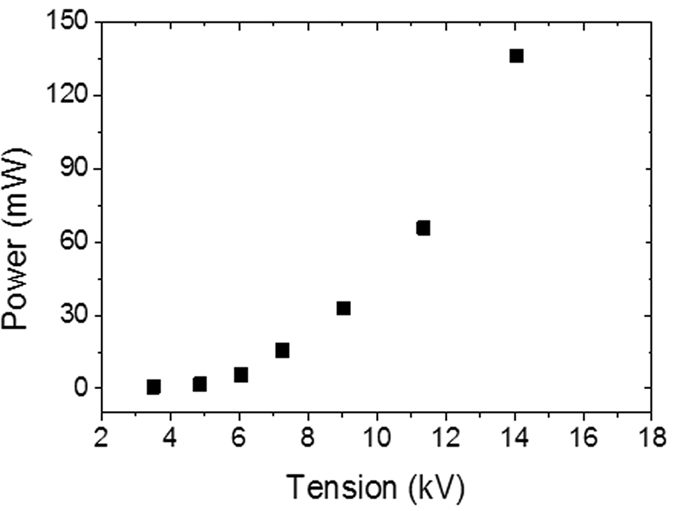
Injected power as a function of applied voltage and constant frequency (50 Hz) under dry air flow at 1 bar.

Figure 4 shows the injected power as a function of applied voltage and constant frequency (50 Hz) under dry air flow at 1 bar.

## Results and Discussions

### Quantitative measurement of adsorbed species using Sorbent track

**Figure 5:**
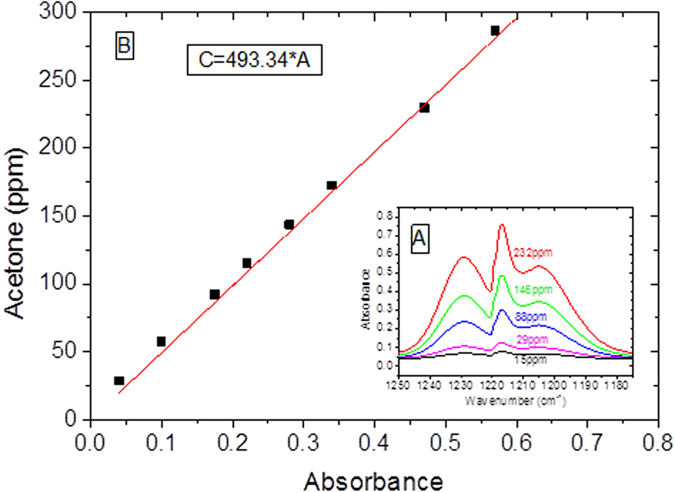
(**a**) Acetone FTIR spectra in the gas phase obtained in a 10 m. White cell inlet acetone concentration in dry air. (**b**) Corresponding acetone calibration curve.

We will estimate the quantity of adsorbed IPA and acetone using the Beer’s Law. In the solid or gaseous state, the Beer’s law express as:


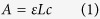


where 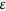 is the absorption coefficient, *L* the pathlength, and *C* the volumic concentration of the sample (mol/L). Acetone and IPA absorbance are first measured in the gas phase using a 10 m White cell in order to correlate absorbance and absolute number of absorbing molecule on the optical path. Calibration curves were obtained by passing the standard gases in air at different known concentrations, through the 10 m White gas cell. The fitting function is shown in Fig. 5. The spectral region selected for acetone calibration is 1207–1197 cm^−1^. A similar calibration is made for IPA in the 992–944 cm^−1^ spectral region.

Experimentally, we find


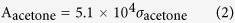


Similarly,


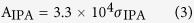


Where σ_acetone_ and σ_IPA_ are the LxC product expressed in mol.cm^−2^. In the following, absolute values of adsorbed concentration will be estimated for IPA and acetone.

### Acetone adsorption and *in situ* plasma oxidation study

#### Adsorption of acetone

200 ppm of acetone diluted in air is sent into the Sorbent-TRACK system, with a total flow rate of 500 mL/min. In Sorbent-TRACK, acetone molecules may be located on the catalyst and in the gas phase. Hence the absorbance is:


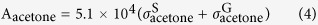


**Figure 6:**
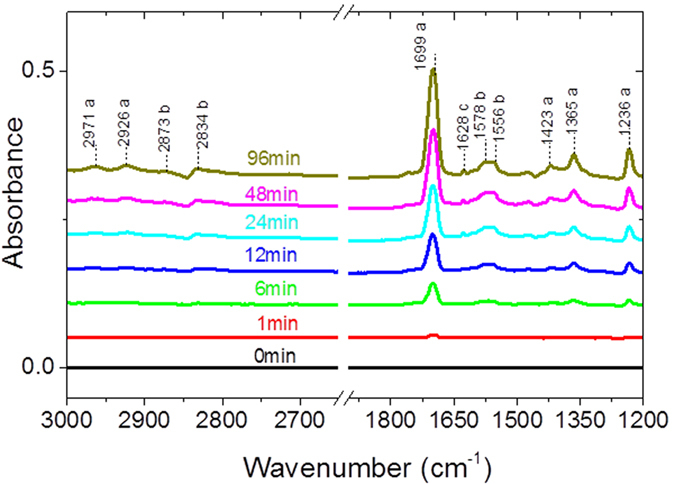
IR absorbance spectra recorded during acetone adsorption on CeO_2_. The spectrum recorded at t = 1 min corresponds mostly to acetone in the gas phase which represents about 3% of acetone concentration at 96 min.

σ^S^_acetone_ + σ Gacetone are the optical depth of acetone in the solid and gaseous phase respectively. In order to evaluate the contribution of the gas phase molecules to the Absorbance in Sorbent-TRACK, the dynamic of absorbance is recorded. Figure 6 shows the infrared spectra of the CeO_2_ surface upon acetone adsorption. Spectra are recorded in the range 3000–1200 cm^−1^ and the clean CeO_2_ spectrum is subtracted to the spectra collected during adsorption. At t = 1 min, as shown in Fig. 6, there is a new band at 1699 cm^−1^. The volume of sorbent-TRACK being 460 ml, the filling time is about 1 min. Hence the spectrum recorded at 1 min by sorbent track is mainly in gas phase. The contribution of gas phase of acetone is about 3% of acetone concentration at 96 min. In the following, the concentration of adsorbed acetone has been corrected from the gas phase contribution.

At t = 96 min, CeO_2_ sample is saturated by acetone. The bands at 2971, 2926, 1699, 1365, and 1236 cm^−1^ are assigned to adsorbed acetone on the CeO_2_ particle surface^23^. These bands are respectively assigned to ν_s-CH3_, ν_as-CH3_, ν_C=O_, δ_s-CH3_ and ν_C-C_ vibration modes of molecularly adsorbed acetone. The bands at 1628 and 1423 cm^−1^, are assigned to *v*_C=O_ and δ_CH_ vibrations in −CH_2-__C=O_ groups of diacetone alcohol-like species^24^. Furthermore, the bands at 1574 cm^−1^ along with the bands at 1554 cm^−1^ are characteristic of the ν_C=O_ and ν_C=C_ vibration modes of mesityl oxide. The different steps of acetone adsorption on CeO_2_ are summarized below^24^.

1. Acetone initially adsorbs on Lewis acid sites of the surface (Ce^4+^):
2. If basic sites (−OH^−^ or O^2−^) are in the vicinity of Ce^4+^ site on which acetone is adsorbed, the C-H bond may be activated for α-hydrogen abstraction and the consequent formation of an anionic enolate-type ion:- Reaction of the enolate species with another acetone molecule to lead to DAA intermediate:- Dehydration of DAA to give adsorbed mesityl oxide and H_2_O_(g)_ or H_2_O_(ads)_.
3. If no basic sites are in the vicinity, acetone remains adsorbed on Lewis site.

**Figure 7:**
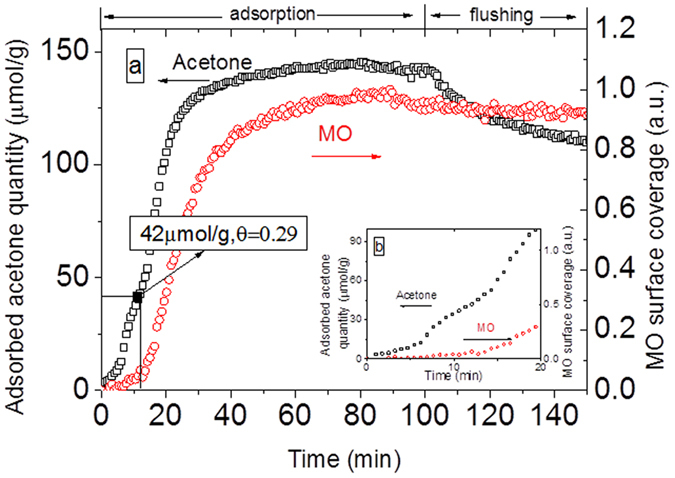
(**a**) Evolution of acetone (gas contribution has been substracted) and mesityl oxide (MO) on the surface of CeO_2_ during the adsorption of acetone and flushing of air under 500 mL/min of air. (for acetone, the amount of acetone adsorbed is 145 μmol/g (θ = 1)); (**b**) a zoom of Fig. 7a between 0 and 20 min.

The temporal evolutions of adsorbed acetone and mesityl oxide during acetone adsorption on the CeO_2_ surface are plotted in Fig. 7 (The contribution of gas phase has been substracted). During the first one minutes, acetone surface coverage on CeO_2_ steeply increases whereas mesityl oxide is not yet observed on the surface, as shown in Fig. 7. This stage correspond the sorbent track system filling and is dominant by gas phase acetone. From 1 min, the acetone surface coverage rate increases while mesityl oxide is still not yet product. As soon as the surface coverage of acetone reaches 0.29 (42 μmol/g), the production of mesityl oxide starts. This behavior suggests that mesityl oxide formation is controlled by a threshold regarding acetone surface coverage. The same behavior is noticed by El Maazawi *et al*.^25^ using the Langmuir adsorption technique and Barakat *et al*.^18^ by DRIFT on TiO_2_ surface. They report a threshold acetone surface coverage of 0.3 and 0.35 respectively, which is consistent with our result. In the adsorption step, the total amount of adsorbed acetone reached 145 μmol/g. When the pollutant is removed, the catalyst is flushed by dry air to remove the reversibly adsorbed acetone which is about 40 μmol/g. The value of acetone at the end of flushing step give the amount of acetone irreversibly adsorbed of 105 μmol/g.

### Adsorbed acetone oxidation by *in-situ* plasma

**Figure 8:**
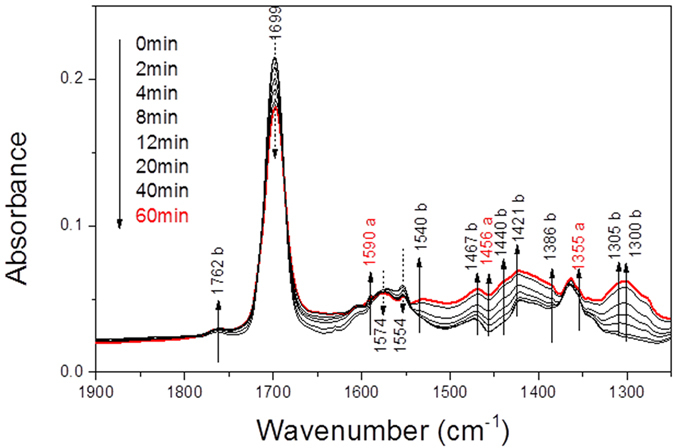
IR absorbance spectra of the evolution of surface species adsorbed on CeO_2_ during *in-situ* plasma (500 mL/min air, 27 mg CeO_2_/KBr, 294 K, 50 Hz, 14 kV, 133 mW).

The infrared spectra obtained upon *in-situ* plasma exposure of acetone saturated CeO_2_ surface are shown in Fig. 8 (applied voltage 14 kV, frequency 50 Hz, injected power 133 mW). Exposing the surface to plasma induced species results in a decrease in the intensities of acetone (1699 cm^−1^ (ν_C=O_)) and of mesityl oxide (1574 cm^−1^ ν_C=O_ and 1554 cm^−1^ ν_C __=__C_ ), related bands which is mentioned above.

Furthermore, the adsorption peaks at 1540, 1440, 1386 and 1305 cm^−1^ are related to the acetate species in the spectra^26^. Whereas those at 1540 and 1440 cm^−1^ are assignable to antisymmetric and symmetric ν_COO−_, the adsorption at 1386 and 1305 cm^−1^ are due to the δ_CH3_ vibrations and the adsorption at 1467 and 1421 cm^−1^ are due to the δ_CH_ vibrations. On the other hand, the absorptions at 1762 and 1300 cm^−1^ can account for ν_C=O_ and δ_OH_ of AcOH molecules. The components at 1590, 1456 and 1355 cm^−1^ suggest the presence of isobutene which is also observed by Rivallin *et al*.^16^. They propose that acetone decomposition only occurs after its aldolization on γ-Al_2_O_3_ into mesityl oxide which fragments into acetaldehyde and isobutene. However, in our case, the formation of acetate surface species is rather favorable. Indeed, the acid-base pair sites (M^n+^ − O^2−^) is available on CeO_2_, but not on Al_2_O_3_. Zaki *et al*.^27^ indicated that strong base sites are necessary for the enolate formation (scheme 1), whereas strong lewis acide sites are essential to stabilize the reaction intermediates. Coordinated acetone molecules may also be activated for a nucleophilic attack on α-carbons, leading to the splitting of a methyl group in form of CH_4,g_ and formation of acetate surface species^26^.


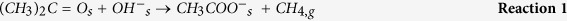


or can be activated for a bi-molecular reaction^28^.


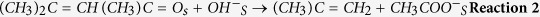


Acetic acid is believed to be formed by the following reaction:


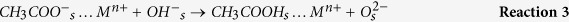


**Figure 9:**
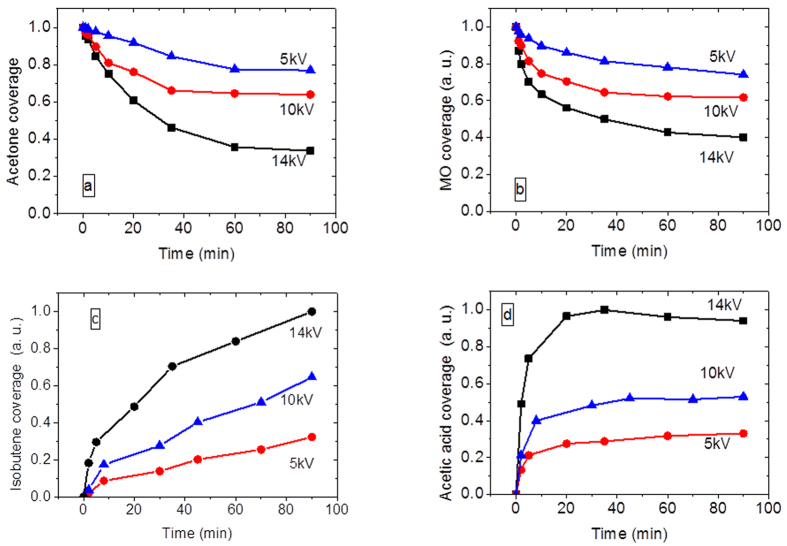
Evolution of acetone (**a**), mesityl oxide (**b**), acetate acid (**c**), isobutene (**d**) on CeO_2_ during *in-situ* plasma exposure at different injected powers: 134 mW, 14 kV, 60 min/40 mW, 10 kV, 60 min/3.1 mW, 5 kV, 60 min.

The temporal evolution of the peaks characteristics of acetone and mesityl oxide and its decomposition products isobutene and acetate acid (Fig. 9) are followed during plasma treatment under pure air at different injected powers. The acetone peak intensities are normalized with respect to the highest intensity of the corresponding experiment (i.e. the intensity of acetone before turning on the plasma) and mesityl oxide, isobutene and acetic acid are reported in arbitrary units. The peaks followed for the species evolution are 1699 cm^−1^ for acetone, 1554 cm^−1^ for mesityl oxide, 1726 cm^−1^ for acetic acid and 1590 cm^−1^ for isobutene.

Data presented in Fig. 9 lead to the following observations:

(i) The acetone and mesityl oxide consumption rate increases with increasing injected power (Fig. 9a,b).)

(ii) The rate of formation of all oxidation products (acetic acid and isopropanol) also increases with increasing injected power (Fig. 9c,d).

(iii) Acetic acid initially accumulates on the surface but is further slightly oxidized as its surface coverage decreases with plasma exposure (Fig. 9c).

(iv) The trend of acetic acid and isobutene on the CeO_2_ surface are not similar (Fig. 9c,d).

Points (i) and (ii) imply that the evolutions of all the adsorbed species are dependent on injected power. In addition, points (iii) and (iv) could imply that the formation reaction pathway is not the same where isobutene is mainly formed by reaction 2 and acetic acid results from reaction 1, 2 and 3.

### Isopropanol adsorption and *in situ* plasma oxidation

#### Adsorption of isopropanol

Similarly to what is discussed in the previous section, during adsorption process, IPA molecules are in gas phase and adsorbed phase. The related absorbance is therefore:


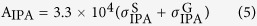


(the gas phase contribution is about 5% according to the method described in section of acetone).

**Figure 10:**
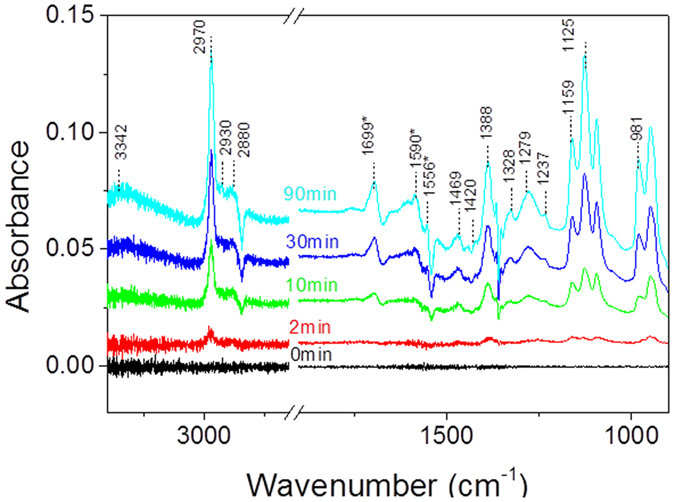
IR spectra recorded by sorbent track during IPA adsorption on CeO_2_ (500 mL/min, air, 28 mg, 100 ppm IPA, 294 K).

Figure 10 shows that IR spectra recorded by sorbent track during IPA adsorption on CeO_2_. The introduction of IPA in the system leads to the appearance of several new absorption bands located at 3342 (broad), 2980, 2930, 2880, 1469, 1420, 1388, 1328, 1279, 1237, 1159, 1125 and 981 cm^−1^. Generally, the bands appearing at 2970, 2930, 2880 cm^−1^ correspond to C-H (ν_CH3_) symmetric and asymmetric stretch modes of the different adsorbed IPA species. These bands are accompanied by symmetric C-H bends (δ_as CH3_) at 1469 cm^−1^, anti-symmetric C-H bends (δ_as CH3_) at 1328 cm^−1^. The skeletal C-C vibration appears at 1237 cm^−1^. Similarly to TiO_2_^18^, the bands at 1159 and 1125 cm^−1^ show two distinct δ_C-O_ vibrations, respectively representative of a dissociative and non dissociative IPA adsorption on CeO_2_. These two adsorption modes are, associated with surface isopropoxide ions, whereas the 981 cm^−1^ band, strongest, originates from intact IPA molecules hydrogen-bonded to the surface. The dissociative adsorption is also supported by the production of OH groups in the high wavenumber range. Indeed, a large band with a maximum at 3342 cm^−1^ corresponding to the OH stretch (ν_OH_) of interacting hydroxyl groups increases with IPA breakthrough on the surface while the bands at 1388 cm^−1^ also indicates strongly non dissociated IPA.

In addition to what was observed on CeO_2_, new peaks appear at 1699, 1590 and 1556 cm^−1^. These peaks are attributed to acetone and its condensation products which have been discussed above.

**Figure 11:**
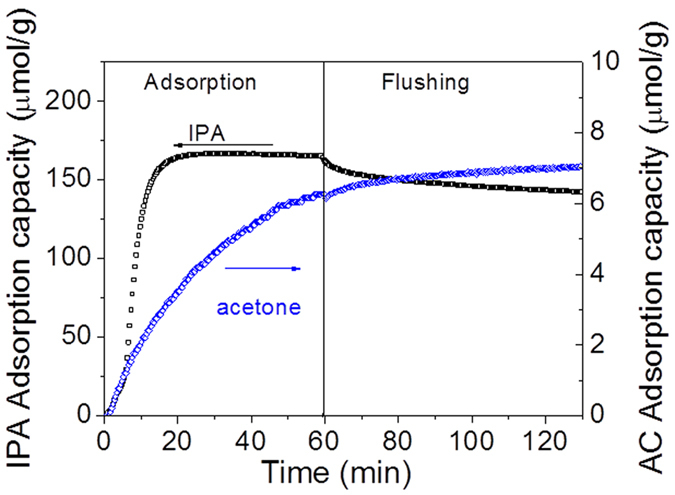
Quantitative evolution of IPA and acetone on CeO_2_ during IPA adsorption and flushing. Acetone results from oxidative adsorption of IPA on ceria.

Figure 11 presents the quantitative evolution of IPA and acetone during the adsorption of 100 ppm of IPA followed by flushing under air. The surface is saturated by IPA (166 μmol/g) from 20 min while the quantity of adsorbed acetone still increases. As the site for IPA is saturated, the new generated acetone should be adsorbed by a different site than IPA. Arsac *et al*. evidenced^29^ that an acetone molecule formed by the photocatalytic oxidation of IPA adsorbed on a “S_1_” site cannot remain adsorbed on the TiO_2_ surface: it must either desorb rapidly as gaseous acetone or diffuse on the surface to be adsorbed on “S_2_” sites, specific to acetone, or on the “S_1_” sites liberated by the removal of IPA surface species during oxidation. The formation of mesityl oxide is not observed because the adsorbed acetone concentration is still low, about 7 μmol/g. It was shown in Fig. 7 that as soon as the surface coverage of acetone reaches 0.29 (45 μmol/g), the production of mesityl oxide starts. The flushing step results in the evacuation of IPA, which amounts to 24 μmol/g.

#### NTP oxidation of isopropanol

**Figure 12:**
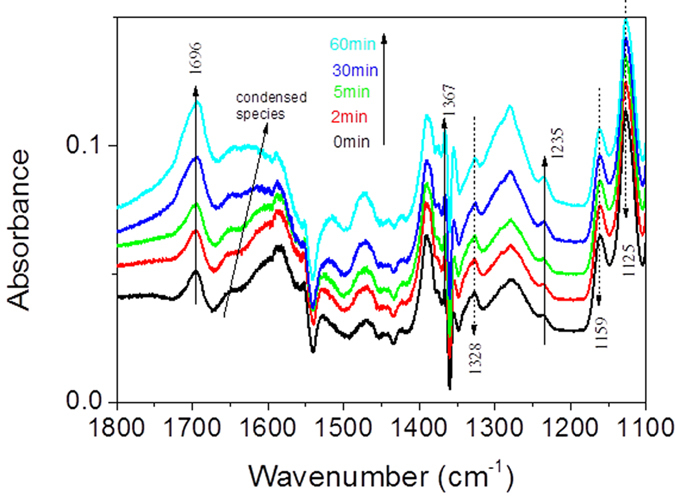
IR absorbance spectra of the evolution of surface species adsorbed on CeO_2_ during *in-situ* plasma (100 ppm IPA, 500 mL/min air, 27 mg CeO_2_/KBr, 294 K, 50 Hz, 9 kV, 32 mW).

The infrared spectra obtained upon *in-situ* plasma exposure of IPA saturated CeO_2_ surface are shown in Fig. 12 (applied voltage 9 kV, frequency 50 Hz, injected power 32 mW). Exposing the surface to the plasma results in a increase in the intensities at 1696, 1367 and 1235 cm^−1^ corresponding to the acetone formation on CeO_2_, concomitantly with disappearance of the previous IR features of the IPA (C-H vibrations (ν_C-H_) in the high wavenumber range and at δ_C-O_ 1159 and 1125 cm^−1^). On the other hand, other broad components appear at 1670–1550 should be contributed to the acetone condensation at CeO_2_ surface, as discussed in section of acetone.

**Figure 13:**
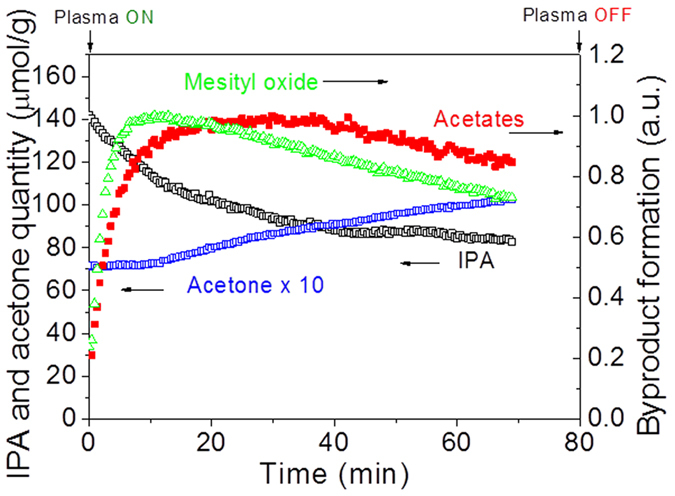
Evolution of IPA and its oxidation products, acetone, mesityl oxide and acetate apparent surface coverage on CeO_2_ during the *in-situ* plasma exposure (500 mL/min air, 50 Hz, 14 kV, 134 mW).

The evolution of IPA and its oxidation products, acetone, mesityl oxide and acetate apparent surface coverage during the *in-situ* plasma exposure is plotted as a function of treatment time in Fig. 13. The initial and rapid consumption of IPA must result from its direct reaction with the oxidative peroxide species induced by plasma to yield acetone and its condensation product, mesityl oxide. Recall from Figs 10 and 11 that the adsorption of IPA on CeO_2_ resulted in the formation of acetone species with the metal oxide. The same phenomenon is believed to take place under plasma exposure, whereby IPA reacts with via adsorbed atomic oxygen to give acetone. In parallel, strongly adsorbed acetone, mesityl oxide and acetates gradually accumulate on the surface at the first 20 min. The surface coverage of the three adsorbed oxidation intermediates decreases with treatment time, implying that the species are oxidized. Adsorbed acetone concentration increases during the 70 min experiments. It comes of course from the IPA oxidation and from the creation of free adsorption sites for acetone during the process. The acetone accumulation is related to the mesityl oxide and acetate decomposition rate and should be the limiting step for IPA decomposition.

## Conclusion

Operando IR spectroscopy was found to be an ideal technique for studying plasma catalytic coupling. Coupling this technique with *in-situ* plasma allowed real-time monitoring of both the pollutant adsorbed on the surface of the catalyst and the by-products produced. The sorbent-TRACK system made in our laboratory provides access to the activity, selectivity and mechanism of the process and could provide a quantitative analysis.

To demonstrate the reliability of this technique, two VOCs were studied in this work: acetone and isopropanol. The dynamic of adsorption of IPA or acetone on ceria followed by their oxidation under plasma exposure has been successfully monitored. Direct adsorption capability was estimated using Trans-FTIR analysis. It was seen that on CeO_2_, Acetone initially adsorbs molecularly on Lewis acid sites (Ce^4+^) of the metal oxide surfaces. When its surface coverage reaches ~30%, two adsorbed acetone molecules react through an aldol condensation to yield mesityl oxide. IPA adsorbs molecularly via hydrogen bonds and by heterolytic dissociation, with a proton going to a surface lattice oxygen and an alkoxide to a surface ion, where the cation acts as a Lewis acid site and the surface oxygen ion acts as a Lewis base.

Both two VOC could be decomposed by *in-situ* plasma, acetone and the condensation species are decomposed into isobutene, acid acetic and gas phase CO_2_. The complete oxidation of the intermediate species (isobutene and acid acetic) occurs via redox reactions with the oxide surface. The oxidation of IPA on CeO_2_ results in adsorbed acetone, mesityl oxides and acetates along with gas phase CO_2_.

## Additional Information

**How to cite this article**: Jia, Z. and Rousseau, A. Sorbent track: Quantitative monitoring of adsorbed VOCs under in-situ plasma exposure. *Sci. Rep.* **6**, 31888; doi: 10.1038/srep31888 (2016).

## References

[b1] Oda T., Kato T., Takahashi T. & Shimizu K. Nitric oxide decomposition in air by using nonthermal plasma processing with additives and catalyst. IEEE Trans. Ind. Appl. 34, 268–272 (1998).

[b2] Thevenet F., Sivachandiran L., Guaitella O., Barakat C. & Rousseau A. Plasma–catalyst coupling for volatile organic compound removal and indoor air treatment: a review. J. Phys. D. Appl. Phys. 47, 224011 (2014).

[b3] Spencer L. F. . CO_2_ dissociation in an atmospheric pressure plasma/catalyst system: a study of efficiency. Plasma Sources Sci. Technol. 22, 015019 (2013).

[b4] Zhu X., Huo P., Zhang Y., Cheng D. & Liu C. Structure and reactivity of plasma treated Ni/Al2O3 catalyst for CO2 reforming of methane. Appl. Catal. B Environ. 81, 132–140 (2008).

[b5] Review of: ‘Indoor Air Pollutants: Exposure and Health Effects’: report on a WHO meeting. EURO Reports and Studies, No. 78. (Copenhagen: WHO Regional Office for Europe, 1983). [Pp.42.] Sw fr 4-0 ISBN-92-890-1244-7. *Ergonomics* **28**, 715–715 (2007).

[b6] Apter A., Bracker A., Hodgson M., Sidman J. & Leung W. Y. Epidemiology of the sick building syndrome. J. Allergy Clin. Immunol. 94, 277–288 (1994). 8077580

[b7] Zhang Y. & Xu Y. Characteristics and correlations of VOC emissions from building materials. Int. J. Heat Mass Transf. 46, 4877–4883 (2003).

[b8] Khan F. I. & Ghoshal K. A. Removal of Volatile Organic Compounds from polluted air. J. Loss Prev. Process Ind. 13, 527–545 (2000).

[b9] Spivey J. J. Complete catalytic oxidation of volatile organics. Ind. Eng. Chem. Res. 26, 2165–2180 (1987).

[b10] Fox M. A. & Dulay M. T. Heterogeneous photocatalysis. Chem. Rev. 93, 341–357 (1993).

[b11] Yamamoto T. . Control of volatile organic compounds by an AC energized ferroelectric pellet reactor and a pulsed corona reactor. IEEE Trans. Ind. Appl. 28, 528–534 (1992).

[b12] Nunez C. M. . Corona destruction: an innovative control technology for VOCs and air toxics. Air Waste 43, 242–247 (1993). 15739519 10.1080/1073161x.1993.10467131

[b13] Parvulescu V. I. Magureanu M. Lukes P. Plasma Chemistry and Catalysis in Gases and Liquids, Wiley-VCH, ISBN-13: 978-3-527-33006-5 (2012).

[b14] Jia Z., Barakat C., Dong B. & Rousseau A. VOCs Destruction by Plasma Catalyst Coupling Using AL-KO PURE Air Purifier on Industrial Scale. J. Mater. Sci. Chem. Eng. 3, 19 (2015).

[b15] Stere C. E. . Probing a Non-Thermal Plasma Activated Heterogeneously Catalyzed Reaction Using *in Situ* DRIFTS-MS. ACS Catal. 5, 956–964 (2015).

[b16] Rivallan M., Fourré E., Aiello S., Tatibouët J.-M. & Thibault-Starzyk F. Insights into the Mechanisms of Isopropanol Conversion on γ-Al2O3 by Dielectric Barrier Discharge. Plasma Process. Polym. 9, 850–854 (2012).

[b17] Sauce S. . New insights in understanding plasma-catalysis reaction pathways: study of the catalytic ozonation of an acetaldehyde saturated Ag/TiO2/SiO2 catalyst. Eur. Phys. J. Appl. Phys. 71, 20805 (2015).

[b18] Barakat C., Gravejat P., Guaitella O., Thevenet F. & Rousseau A. Oxidation of isopropanol and acetone adsorbed on TiO2 under plasma generated ozone flow: Gas phase and adsorbed species monitoring. Appl. Catal. B Environ. 147, 302–313 (2014).

[b19] Jia Z. . Acetaldehyde removal using a diphasic process coupling a silver-based nano-structured catalyst and a plasma at atmospheric pressure. Catal. Today, http://dx.doi.org/10.1016/j.cattod.2012.10.028 (2013).

[b20] Jia Z. . Growth of Silver Nanoclusters on Monolayer Nanoparticulate Titanium-oxo-alkoxy Coatings. J. Phys. Chem. C 116, 17239–17247 (2012).

[b21] Trovarelli A. & Fornasiero P. Catalysis by ceria and related materials. at http://cds.cern.ch/record/1606148 (2013).

[b22] Manley T. C. The Electric Characteristics of the Ozonator Discharge. Trans. Electrochem. Soc. 84, 83 (1943).

[b23] Panov A. & Fripiat J. J. An Infrared Spectroscopic Study of Acetone and Mesityl Oxide Adsorption On Acid Catalyst. 7463, 3788–3796 (1998).

[b24] Zaki M. I., Hasan M. A. & Pasupulety L. Surface Reactions of Acetone on Al2O3, TiO2, ZrO2, and CeO2: IR Spectroscopic Assessment of Impacts of the Surface Acid−Base Properties. Langmuir 17, 768–774 (2001).

[b25] El-Maazawi M. A and Photocatalytic Oxidation of Acetone on TiO2: An *in Situ* Transmission FT-IR Study. J. Catal. 191, 138–146 (2000).

[b26] Hasan M. A., Zaki M. I. & Pasupulety L. Oxide-catalyzed conversion of acetic acid into acetone: an FTIR spectroscopic investigation. Appl. Catal. A Gen. 243, 81–92 (2003).

[b27] Zaki M. I., Hasan M. A., Al-Sagheer F. A. & Pasupulety L. Surface Chemistry of Acetone on Metal Oxides: IR Observation of Acetone Adsorption and Consequent Surface Reactions on Silica−Alumina versus Silica and Alumina. Langmuir 16, 430–436 (2000).

[b28] Zaki M. An infrared spectroscopic study of the adsorption and mechanism of surface reactions of 2-propanol on ceria. J. Catal. 80, 114–122 (1983).

[b29] Arsac F., Bianchi D., Chovelon J. M., Ferronato C. & Herrmann J. M. Experimental microkinetic approach of the photocatalytic oxidation of isopropyl alcohol on TiO2. Part 1. Surface elementary steps involving gaseous and adsorbed C3HxO species. J. Phys. Chem. A 110, 4202–4212 (2006). 16553371 10.1021/jp055342b

